# Aridity Threshold Induces Abrupt Change of Soil Abundant and Rare Bacterial Biogeography in Dryland Ecosystems

**DOI:** 10.1128/msystems.01309-21

**Published:** 2022-02-08

**Authors:** Haibo Pan, Hang Gao, Ziheng Peng, Beibei Chen, Shi Chen, Yu Liu, Jie Gu, Xiaorong Wei, Weimin Chen, Gehong Wei, Shuo Jiao

**Affiliations:** a State Key Laboratory of Crop Stress Biology in Arid Areas, Shaanxi Key Laboratory of Agricultural and Environmental Microbiology, College of Life Sciences, Northwest A&F University, Yangling, People’s Republic of China; b Interdisciplinary Research Center for Soil Microbial Ecology and Land Sustainable Productivity in Dry Areas, College of Natural Resources and Environment, Northwest A&F University, Yangling, People’s Republic of China; c State Key Laboratory of Soil Erosion and Dryland Farming on the Loess Plateau, Northwest A&F University, Yangling, People’s Republic of China; Institute of Soil Science, Chinese Academy of Sciences

**Keywords:** aridity threshold, terrestrial ecosystems, biogeography, microbial diversity, community assembly, microbial co-occurrence, niche conservatism

## Abstract

Aridity, which is increasing worldwide due to climate change, affects the biodiversity and functions of dryland ecosystems. Whether aridification leads to gradual (or abrupt) and systemic (or specific) changes in the biogeography of abundant and rare microbial species is largely unknown. Here, we investigated stress-adaptive changes (aridity-driven, ranging from 0.65 to 0.94) and biogeographic patterns of abundant and rare bacterial communities in different habitats, including agricultural field, forest, wetland, grassland, and desert, in desert oasis transition zones in northern China. We observed abrupt changes at the breakpoint of aridity values (0.92), characterized by diversity (α-diversity and β-diversity), species coexistence, community assembly processes, and phylogenetic niche conservatism. Specifically, when aridity was <0.92, increasing aridity led to more deterministic assembly and species coexistences for the abundant subcommunity, whereas the reverse was observed for the rare subcommunity. The phylogenetic niche conservatism for both subcommunities increased slowly with aridity. When aridity was >0.92, the systemic responses of abundant and rare taxa changed dramatically in a consistent direction, such that both subcommunities rapidly tended to have a more deterministic assembly, species coexistence, and stronger phylogenetic niche conservatism with increasing aridity. In addition, the change rates of abundant taxa were higher than those of rare taxa, indicating the more sensitive responses of abundant taxa along aridity variation. This finding has important implications for understanding the impact of aridity on the structure and function of abundant and rare soil taxa and how diversity maintenance is associated with soil microbiota responding to global change. The abrupt threshold of soil bacteria found can be used for buffering and for building effective adaptation and mitigation measures aimed at maintaining the capacity of drylands for basic ecosystem functioning.

**IMPORTANCE** Aridity, which is increasing worldwide due to climate change, affects the biodiversity and functions of dryland ecosystems. We provided the first statistical evidence for abrupt changes of species coexistence, ecological processes, and niche conservation of abundant and rare soil bacteria triggered by diversity to abrupt increases in aridity. The abrupt threshold of soil bacterial community response to aridity is spatially heterogeneous at the local scale and should be specified according to local conditions for buffering and for building effective adaptation and mitigation measures aimed at maintaining the capacity of drylands for basic ecosystem functioning.

## INTRODUCTION

Research on microbial biogeography is indispensable to deciphering the mechanisms that generate and maintain microbial diversity and to predict how soil processes respond to climate change (1–3). Greater environmental heterogeneity across a large spatial scale could form different ecological niches, enabling the coexistence of microorganisms with distinct life strategies (4, 5). This environmental heterogeneity can also generate a strongly skewed abundance distribution within the indigenous microbial community (6, 7), with a small number of abundant species and a large number of rare species (8–11). Understanding the biogeographic and ecological assembly of the rare and abundant subcommunities is essential for predicting microbe-driven ecosystem processes and functions.

Drylands, areas characterized by aridity (1-AI; 1-mean annual precipitation [MAP]/mean annual potential evapotranspiration) values of >0.65 (12), cover more than 40% of the terrestrial surface (13) and are highly vulnerable to human activities, climate change, and land degradation (14, 15). Global changes are predicted to exacerbate processes leading to a further increase the total area of drylands globally (16). Increasing aridity is a major force of climate change in global drylands (14) and affects multiple ecosystem biodiversity and functional attributes (e.g., species richness, abundance, geographic patterns, and their interactions with abiotic factors) (17). It is crucial to clarify whether their responses to aridity intensification are gradual or abrupt (15, 17, 18). Recent studies have indicated that multiple ecosystem structures and functions present two stages of abrupt change with the intensification of aridity, namely, the soil disruption phase (aridity of >0.7), with declines in organic carbon, total nitrogen and clay contents, stability of aggregates, and relative abundance of fungal functional groups (15, 19), and the ecosystem breakdown phase (aridity of >0.8), with extreme reductions in plant cover and exponential increases in albedo (15, 20). However, we still have limited knowledge on whether increases in aridity lead to abrupt changes in the biogeography of the abundant and rare subcommunities.

Disentangling the mechanisms underlying microbial community assembly and species coexistence is a central issue in microbial ecology (11, 21–24). Community assembly is jointly shaped by deterministic (e.g., variable selection and homogeneous selection) and stochastic (e.g., dispersal limitation and homogenizing dispersal) processes (8, 25, 26). These assembly processes could also influence species coexistence. For example, deterministic processes (the selection of abiotic and biotic factors) provide distinct, diverse niches (27) and stochastic processes (random birth, death, and dispersal events) allow species to co-occur with considerable overlap of niches by closely matching competitive capacities and unrelated random events with environmental variation (28). The dynamic balance of the two ecological processes, regulated by environmental factors (e.g., pH, salinity, sulfur, and mean annual temperature [MAT]), mediates microbial coexistence and species composition (8, 10, 24, 29, 30). Despite this knowledge, our understanding of whether aridity mediates the balance between stochasticity and determinism in community assembly of rare and abundant taxa, and how ecological processes influence microbial coexistence in dryland ecosystems, remains limited.

Phylogenetic distribution of microbial functional traits could aide in the prediction of microbial community response to global change (31). Despite the promiscuity of horizontal gene transfer among microbes, microbial responses to environmental change appear to be phylogenetically conserved (32–35). A study of multifactor perturbations showed that the depth of the clades conserved within bacterial communities across locations responded uniformly to environmental change (31). However, it remains unknown whether phylogenetic niche conservation of bacterial responses to environmental gradients exhibit abrupt changes with increasing aridity, particularly for abundant and rare taxa.

Here, we aimed to answer whether biogeography and community assembly of abundant and rare soil bacteria exhibited nonlinear responses with increasing aridity. We addressed this question using the high-throughput sequencing data sets (36, 37) of soil bacteria from a large-scale survey, covering agricultural field, forest, wetland, grassland, and desert along the Hexi Corridor (transect intervals of 1,257.6 km) in the northwest arid region of China. Our study identified the aridity threshold (aridity value of 0.92) for the abrupt changes in bacterial diversity, species coexistence, community assembly, phylogenetic niche conservatism, and microbial community potential functionalities with increasing aridity and found distinct response patterns between abundant and rare taxa.

## RESULTS

### General distribution of rare and abundant subcommunities under aridity gradient.

**FIG 1:**
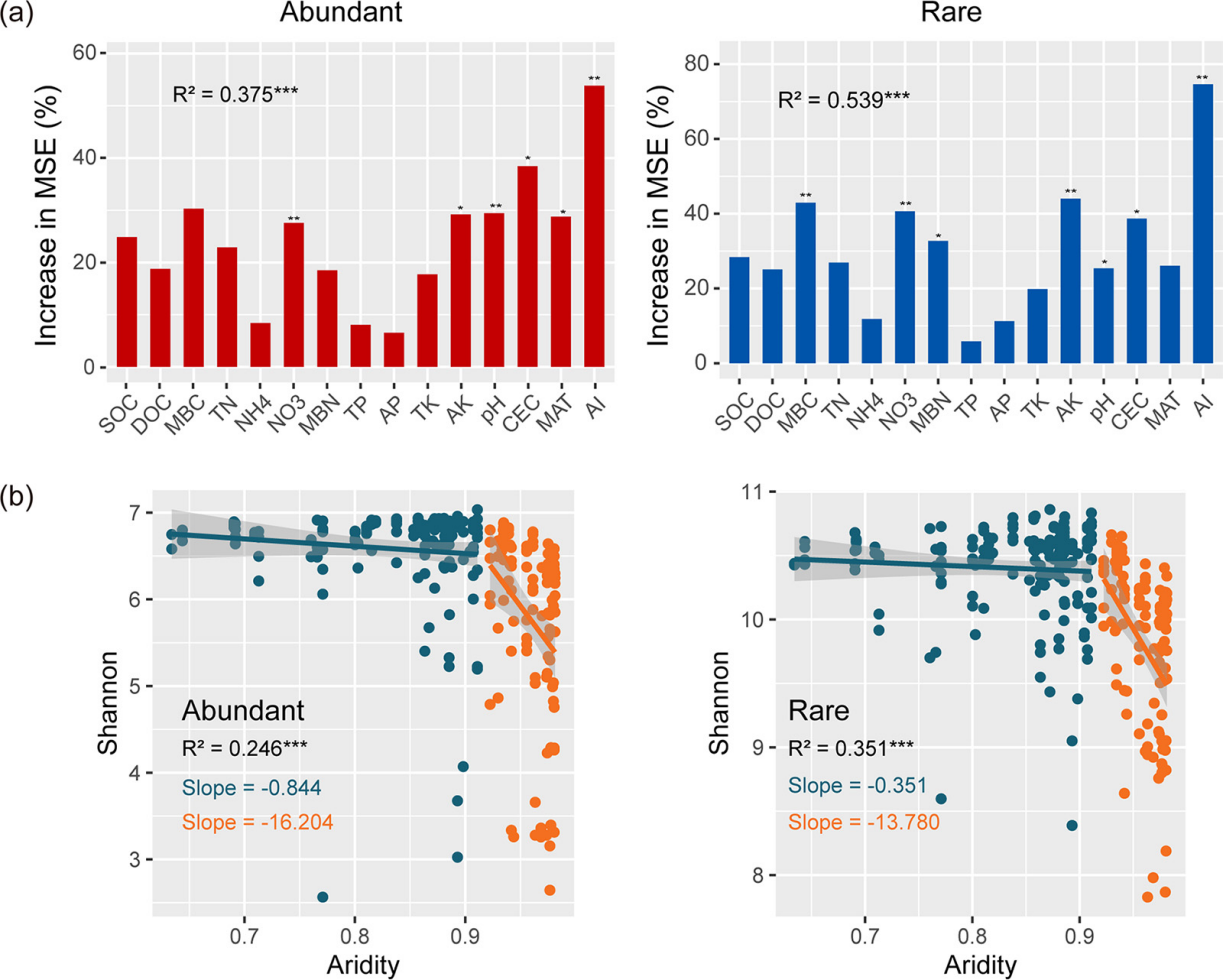
Nonlinear responses of α diversity of abundant and rare subcommunities to aridity in dryland ecosystem. (a) Random forest model identifies the major contributors of α diversity for abundant and rare subcommunities. (b) Segmented regressions of α diversity of abundant and rare subcommunities with increasing aridity. Boxplots indicate the variation in α diversity on either side of the breakpoint of aridity. Asterisks denote significant differences (*, *P < *0.05; **, *P < *0.01; ***, *P < *0.001; Wilcoxon rank-sum test). Shaded areas denote the 95% confidence interval of the regression lines.

As expected, abundant taxa constituted a relatively low proportion of operational taxonomic units (OTUs) (mean, 1.16%) but accounted for 53.5% of the average relative abundance in each sample. Conversely, rare taxa constituted a high proportion of the OTUs (mean, 55.4%), while they contributed to an average of only 12.4% of the relative abundance in each sample. To disentangle the potential main contributors of α-diversity of abundant and rare taxa in terrestrial ecosystems, we applied random forest (RF) analysis (Fig. 1a). The results showed that aridity was the most important variable for explaining the α-diversity. Strong negative correlations between the α-diversity and aridity were observed in multiple habitats, with the exception of wetland (see Fig. S1 in the supplemental material).

10.1128/mSystems.01309-21.1 FIG S1 Heatmaps of correlation (Spearman’s) coefficients between microbial α-diversity all individual soil and climate factors of abundant and rare subcommunities in different habitats and soil depths. The shading from white to red represents low-to-high positive correlation, while the shading from white to blue represents low-to-high negative correlation. *, *P < *0.05; **, *P < *0.01; ***, *P < *0.001. Download FIG S1, TIF file, 1.2 MB. Copyright © 2022 Pan et al. 2022 Pan et al. https://creativecommons.org/licenses/by/4.0/ This content is distributed under the terms of the Creative Commons Attribution 4.0 International license.

We observed that the changes in bacterial α-diversity for abundant and rare taxa with aridity exhibited a nonlinear trend (Fig. 1b). Specifically, once the aridity threshold value (0.92) was reached, small increases in aridity led to drastic decline in the value of the α-diversity, with a greater rate of decline for abundant taxa than rare taxa. Moreover, the change of α-diversity in desert soils was faster than that in other habitats, except for rare taxa in wetland at aridity levels of >0.92 (Fig. S2a). It is worth noting that the α-diversity in agricultural fields increased with increases in aridity under the threshold value of <0.92 (Fig. S2a). In addition, the decline rate of α-diversities in topsoil (depth, 0 to 15 cm) were slower than that in subsoil (depth, 15 to 30 cm) for both abundant and rare taxa at aridity values of >0.92 (Fig. S1b). Given the clear shift in α-diversity at an aridity level of 0.92 for both abundant and rare taxa, we divided all samples into two clusters, lower aridity stress (aridity of <0.92) and higher aridity stress (aridity of >0.92), to evaluate whether there were significant abrupt changes of biogeographic and ecological community assembly with aridity for abundant and rare taxa.

10.1128/mSystems.01309-21.2 FIG S2 Aridity-driven patterns of α diversity of abundant and rare subcommunities in different habitats and soil depths. Asterisks denote significant differences (***, *P < *0.001). Download FIG S2, TIF file, 2.2 MB. Copyright © 2022 Pan et al. 2022 Pan et al. https://creativecommons.org/licenses/by/4.0/ This content is distributed under the terms of the Creative Commons Attribution 4.0 International license.

### Phylogenetic patterns of abundant and rare community composition.

**FIG 2:**
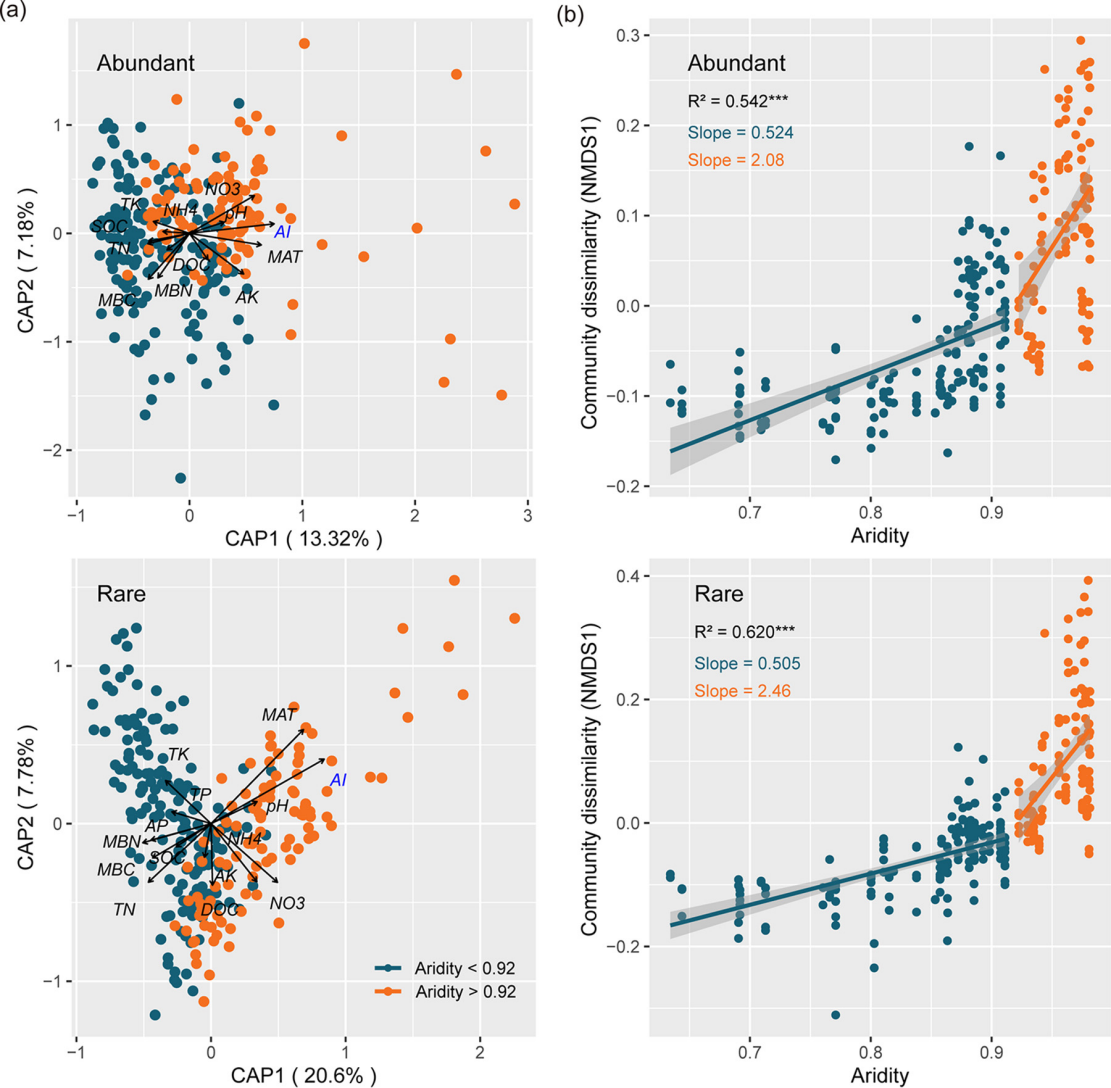
Breakpoint-based microbial β diversity patterns of abundant and rare subcommunities to aridity in dryland ecosystem. (a) Constrained analysis of principal coordinates (CAP) was used to identify major environmental factors based on MNTD metric (βMNTD) in abundant and rare taxa. Gray words represented the environmental variables after collinearity was removed, and blue words were identified as the most influential factors in community structure. (b) Piecewise regression showing community dissimilarity (NMDS1) based on βMNTD to increasing aridity. Asterisks denote significant correlation (***, *P < *0.001). Shaded areas denote the 95% confidence interval of the regression lines.

A constrained analysis of principal coordinates (CAP) based on MNTD metric (βMNTD) indicated that aridity had the strongest effects on the community structure of abundant and rare taxa (Fig. 2a). The correlation (Spearman’s) coefficients were calculated to assess the main phylogenetic β-diversity-driven factor, which was also shown to be positively correlated with aridity (Fig. S3). Likewise, the changes in bacterial community dissimilarity (NMDS1) of abundant and rare taxa with aridity exhibited a nonlinear response at the aridity threshold of 0.92 (Fig. 2b). Furthermore, the NMDS1 change rate of abundant taxa was higher than that of rare taxa (aridity < 0.92), but when the aridity level was over 0.92, the NMDS1 change rate of abundant and rare taxa showed the opposite trend. Nonmetric multidimensional scaling (NMDS) based on βMNTD analysis showed the significant differentiations of phylogenetic β-diversity for abundant and rare subcommunities in different habitats (Adonis, *P < *0.001) (Fig. S4a) and soil depths (only in rare subcommunity) (Adonis, *P < *0.01) (Fig. S4b) on either side of the 0.92 aridity threshold. In particular, the increase of NMDS1 in desert soils was faster than that in other habitats along the aridity gradients, except for abundant taxa in forest at aridity levels of <0.92 (Fig. S5a). The increase of NMDS1 in topsoil was higher than that in subsoil both for abundant and rare taxa undergoing lower aridity stress. However, the increase of community dissimilarity (NMDS1) in topsoil was more rapid than that in subsoil for abundant taxa, while the opposite trend was found in rare taxa under higher aridity stress (Fig. S5b). Additionally, significant but weak distance–decay relationships (DDRs) were observed for the abundant and rare taxa along the Hexi Corridor (*P < *0.05) (Fig. S4c).

10.1128/mSystems.01309-21.3 FIG S3 Heatmaps of correlation (Spearman’s) coefficients between β-diversity for all individual soil and climate factors of abundant and rare subcommunities in different habitats and soil depths. The shading from white to red represents low-to-high positive correlation, while the shading from white to blue represents low-to-high negative correlation. *, *P < *0.05; **, *P < *0.01; ***, *P < *0.001. Download FIG S3, TIF file, 1.3 MB. Copyright © 2022 Pan et al. 2022 Pan et al. https://creativecommons.org/licenses/by/4.0/ This content is distributed under the terms of the Creative Commons Attribution 4.0 International license.

10.1128/mSystems.01309-21.4 FIG S4 β-Diversity trends under different habitats and soil depths of abundant and rare subcommunities. Nonmetric multidimensional scaling (NMDS) based on MNTD metric (βMNTD) analysis results showing the differentiations of β-diversity for abundant and rare subcommunities at an aridity level of 0.92 in different habitats (a) and soil depths (b). (c) The distance–decay relationships (DDRs) based on βMNTD between community similarity of abundant and rare subcommunities. The similarities of communities were tested by Adonis. ***, *P < *0.001. Download FIG S4, TIF file, 2.9 MB. Copyright © 2022 Pan et al. 2022 Pan et al. https://creativecommons.org/licenses/by/4.0/ This content is distributed under the terms of the Creative Commons Attribution 4.0 International license.

10.1128/mSystems.01309-21.5 FIG S5 Changes of community dissimilarity (NMDS1) of abundant and rare taxa along aridity gradients in different habitats and soil depths. ***, *P < *0.001. Download FIG S5, TIF file, 2.9 MB. Copyright © 2022 Pan et al. 2022 Pan et al. https://creativecommons.org/licenses/by/4.0/ This content is distributed under the terms of the Creative Commons Attribution 4.0 International license.

### Assembly processes and species coexistence in abundant and rare subcommunities.

**FIG 3:**
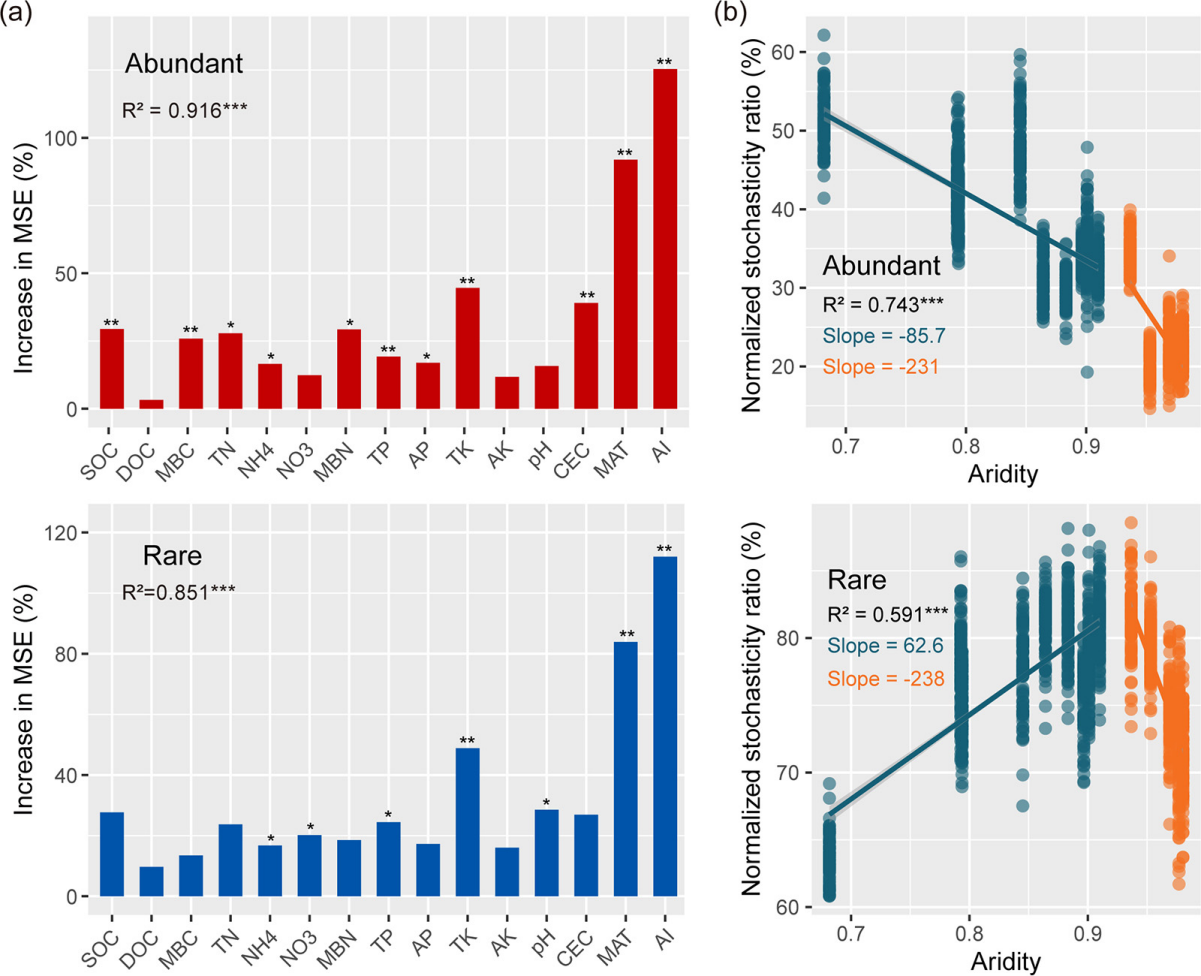
Sequence of abrupt relative influence of deterministic and stochastic assembly processes of abundant bacterial taxa in dryland ecosystems as aridity increases. (a) Random-forest model predicts the main factors for the assembly of both abundant and rare subcommunities. (b) Nonlinear regression models show the relationships of normalized stochasticity ratio (NST) between the abundant and rare bacterial taxa and aridity. NST value (ranging from 0% to 100%) below the 50% boundary point (NST < 50%) represents more deterministic assembly; NST value above the 50% boundary point (NST > 50%) denotes more stochastic assembly. Asterisks denote significant differences (***, *P < *0.001). Shaded areas denote the 95% confidence interval of the regression lines.

The ecological processes shaping bacterial community assembly were explored using composition (normalized stochasticity ratio, NST) based on null-model analysis. RF analysis showed that aridity was identified as the most important contributor to the assembly of both the abundant and rare subcommunities (Fig. 3a). The relationship of assembly processes for abundant and rare taxa to aridity clearly separated into two phases either side of an aridity value of 0.92 (Fig. 3b). The relative influence of stochastic assembly processes decreased significantly in abundant taxa with increasing aridity, while the opposite trend was observed for rare taxa at aridity levels of <0.92. Once this aridity threshold was crossed (aridity of >0.92), there was a sharp strengthening in the relative influence of deterministic assembly for both abundant and rare taxa with the aggravation of aridity stress.

**FIG 4:**
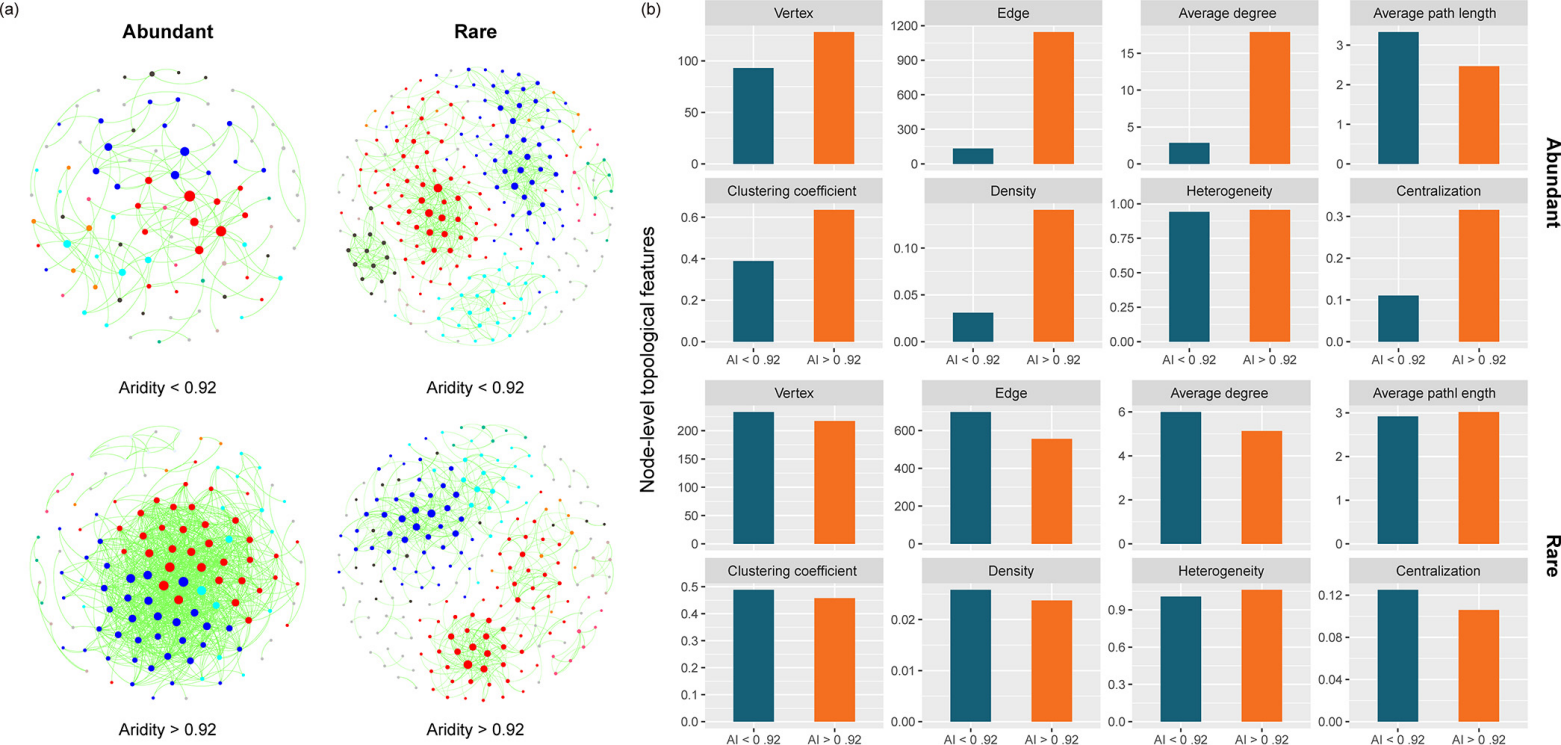
Co-occurrence patterns of abundant and rare taxa at both ends of the aridity threshold. (a) Metacommunity co-occurrence network of abundant and rare taxa on either side of the threshold of aridity. (b) Node-level topological features of abundant and rare taxa at both sides of the aridity threshold, specifically the vertex, edge, average degree, average path length, clustering coefficient, density, diameter, heterogeneity, and centralization.

Given the previous phase transitions, we then built metacommunity co-occurrence networks based on correlation relationships to estimate whether species coexistence patterns of abundant and rare taxa exhibited abrupt changes with increasing aridity. The network analyses showed that higher aridity stress (aridity of >0.92) led to a more complex network for abundant taxa but less obvious changes for rare taxa (Fig. 4a). We found that node-level topological features, including vertex, edge, average degree, clustering coefficient, density, and centralization, increased, while the average path length and heterogeneity decreased for abundant taxa experiencing higher aridity stress (Fig. 4b). In contrast, the topological parameters of rare taxa showed the opposite trends for the above-described features. Moreover, all samples could be divided into 14 clusters on average, and the corresponding local metacommunity co-occurrence networks were constructed to verify whether the topological parameters displayed an abrupt change with increasing aridity. The results showed that the topological parameters responded in a nonlinear manner for both abundant and rare taxa to increasing aridity (Fig. S6). At an aridity value of <0.92, we found that average path length and heterogeneity decreased, clustering coefficient and density of the abundant network increased with the increase in aridity, and the variation trends of corresponding parameters were opposite for rare taxa. However, an abrupt shift in the slope of the relationship between the increase in aridity and topological features, from opposite to consistent, was observed at an aridity value of >0.92 for both abundant and rare taxa.

10.1128/mSystems.01309-21.6 FIG S6 Piecewise regression fitting of network topological parameters with increasing aridity in 14 average clusters of all samples of abundant and rare subcommunities. Solid lines in green and orange represent the fitted linear OLS model with aridity values of <0.92 and >0.92, respectively. The dashed line represents the potential trends between topological features and increasing aridity fitted by locally weighted regression (Loess) under the aridity gradient. *, *P < *0.05; **, *P < *0.01; ***, *P < *0.001. Download FIG S6, TIF file, 2.4 MB. Copyright © 2022 Pan et al. 2022 Pan et al. https://creativecommons.org/licenses/by/4.0/ This content is distributed under the terms of the Creative Commons Attribution 4.0 International license.

### Phylogenetic niche conservatism and potential functional redundancy in abundant and rare subcommunities.

**FIG 5:**
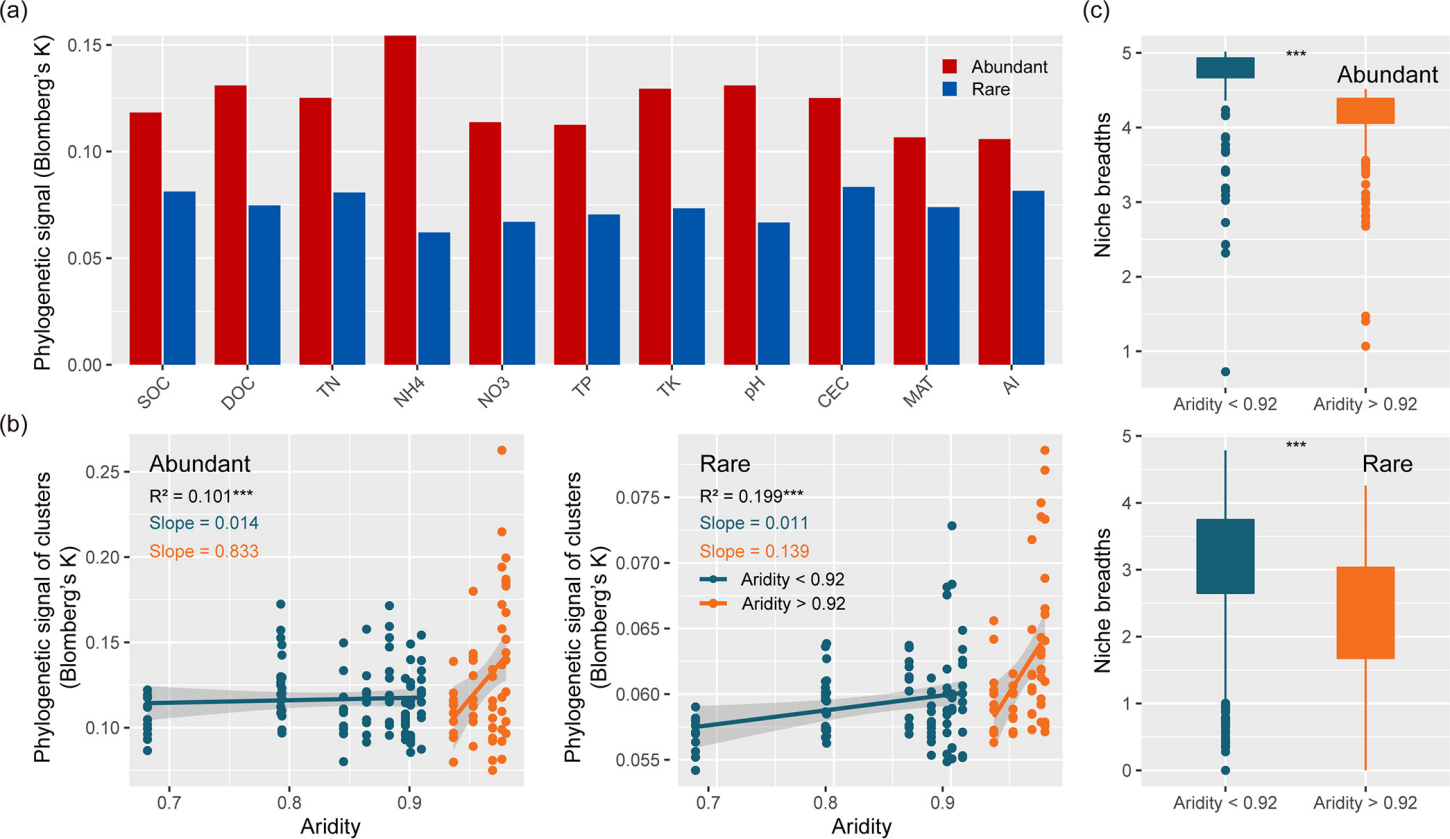
Phylogenetic niche conservatism of abundant and rare taxa along the aridity gradient. (a) Phylogenetic signals of abundant and rare taxa revealing the trait conservatism for environmental preferences were evaluated using Blomberg’s K statistic. (b) Piecewise fitting of Blomberg’s K of all variables under average grouping of samples under aridity gradient. (c) Boxplot shows niche breadths of abundant and rare taxa on either side of the breakpoint of aridity. Asterisks denote significant differences (***, *P < *0.001; Wilcoxon rank-sum test). Asterisks denote significant differences (***, *P < *0.001). Shaded areas denote the 95% confidence interval of the regression lines.

Blomberg's K statistic revealed that the abundant taxa presented stronger phylogenetic signals for all environmental variables (e.g., soil factors and climate factors) compared with the corresponding rare taxa, confirming their higher level of trait conservatism (Fig. 5a). As noted above, we further explored whether there were abrupt changes of Blomberg's K for abundant and rare taxa with increasing aridity. The observed responses of Blomberg's K to increases in aridity revealed weak and strong positive correlations with lower and higher aridity stress for both abundant and rare taxa, respectively (Fig. 5b). The responses suggest that phylogenetic niche conservatism also showed abrupt changes in response to intensified aridity. Additionally, significantly lower niche breadth under lower aridity stress was observed than that of under higher aridity stress for both abundant and rare subcommunities (Wilcoxon rank-sum test, *P < *0.001) (Fig. 5c).

**FIG 6:**
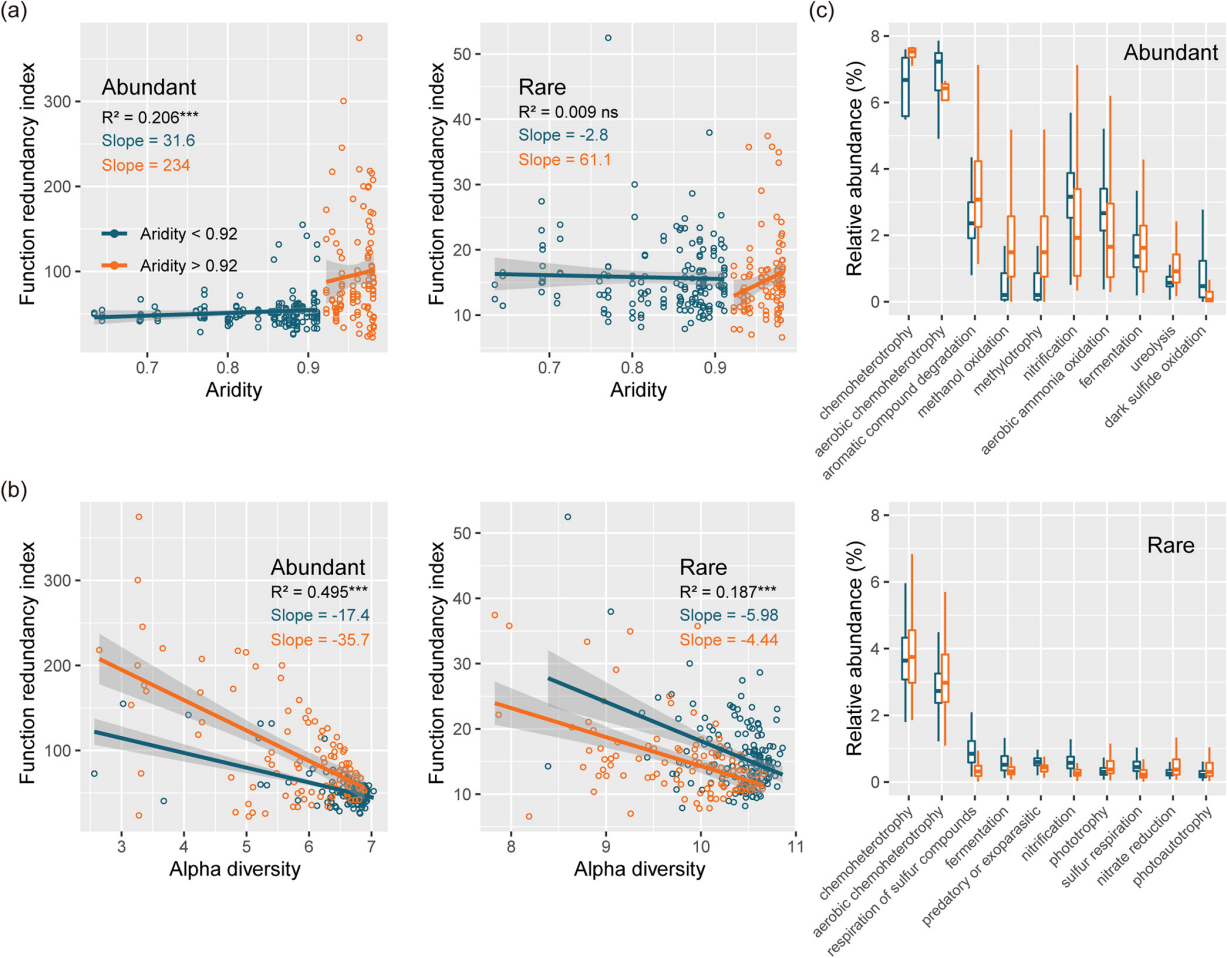
Nonlinear changes of relationships between biodiversity with aridity and function redundancy index of abundant and rare taxa along aridity gradients. (a) The effect of the increasing aridity on the function redundancy index of abundant and rare taxa. (b) The relationships between α diversity and function redundancy index of abundant and rare taxa. Shaded areas denote the 95% confidence interval of the regression lines. (c) The changes of potential functionalities (the top 10 most dominant functionalities) of microbial community at both sides of the aridity threshold were explored with FARPROTAX for abundant and rare taxa.

We further examined the relationships between biodiversity and ecosystem function redundancy index (FDR) of abundant and rare taxa revealed a positive abrupt change along aridity gradients, but only that of abundant taxa was significant (Fig. 6a). Strong negative relationships between α-diversity and FDR were observed both for abundant and rare taxa along the aridity gradients (Fig. 6b). Importantly, our results showed that the slope of diversity-FDR undergoing higher aridity was steeper than that of undergoing lower aridity for abundant taxa. However, the slopes of diversity-FDR for rare taxa for both aridity intensities were similar. Finally, the differences of potential functionalities (the top 10 most dominant functionalities) of microbial community on both sides of the aridity threshold were explored with FARPROTAX for abundant and rare taxa (Fig. 6c). The relative abundance of different functions in abundant taxa was generally higher than that in rare taxa. The functions of both chemoheterotrophy and aerobic chemoheterotrophy were significantly enriched in abundant and rare taxa with increasing aridity.

## DISCUSSION

Understanding the interrelated responses of biodiversity, ecological processes, and niche conservatism to increasing aridity is crucial for improving forecasts of ecosystem responses to climate change. There is modeling and empirical evidence that multiple ecosystem functional and structural characteristics undergo abrupt discontinuous transitions and follow a series of sequential ecological thresholds in relation to increases in aridity (15, 20, 38). For instance, MAP causes a rapid shift in the diversity and structure of soil microbes, which would trigger changes in microbial interactions and assembly processes (39). However, few large-scale studies have quantified whether biogeography distribution and ecological assembly of abundant and rare soil bacteria exhibit abrupt changes to increases in aridity in dryland ecosystems. As far as we know, we provided the first statistical evidence for abrupt changes of species coexistence, ecological processes, and niche conservation of abundant and rare soil bacteria triggered by diversity to abrupt increases in aridity. We demonstrated that (i) the tipping point that triggers the abrupt loss of diversity and functionality in the soil bacterial community of abundant and rare taxa for the dryland ecosystem is an aridity value of approximately 0.9; and (ii) abundant taxa showed sharper changes in responding to aridity stress than rare taxa, with distinct response patterns between abundant and rare taxa.

### Abrupt changes in the diversity of abundant and rare taxa.

Our results showed that soil bacterial α-diversity of abundant and rare taxa responded to increases in aridity in a nonlinear manner, consistent with previous studies that investigated on broader scales (40–42). The nonlinear distribution of bacterial α-diversity with aridity index along a 3,700-km transect of grassland ecosystem in northern China was reported by Wang et al. (43). In their research, they also found that the inflection point of α-diversity change was close to 0.9. The increase of aridity directly reflected the decrease in water and nutrient availability to soil microorganisms (13) and induced the abrupt decline of soil nutrient content (19), which may lead to the decrease of microorganisms adopting eutrophic life strategies (K-strategies) (44). Moreover, poor soil, with decreasing niche dimensionality, can lead to a nonlinear pattern of soil microbial α-diversity loss (45). Specifically, we found that α-diversity in agricultural fields increased with increasing aridity under lower aridity stress (aridity of <0.92). Agriculture is an artificial ecosystem, and the disturbance type and frequency are higher than those in natural ecosystems (e.g., forest, grass, desert, and wetland). Therefore, the disturbance pressure of less aridity was an intermediate disturbance (46) to the agricultural soil bacterial community, which explained the increased α-diversity in this ecosystem under lower aridity stress. In addition, the effects of aridity on microbial biodiversity were stronger in subsoils than in topsoils undergoing higher aridity stress for both abundant and rare taxa. This trend is probably because aridity can directly increase the stress experienced by microorganisms and that the spatial separation of decomposer and substrate appears to play a more important role within the subsoil (41, 47).

In the dryland ecosystem, weak correlation of phylogenetic DDRs, but strong correlation between community dissimilarity and increasing aridity, were observed for both abundant and rare taxa, indicating microclimatic heterogeneity caused by aridity outweighs the effect of spatial isolation on microbial diffusion limitation. Our results showed that community dissimilarity also exhibited a segmented increase pattern with increasing aridity. The nonlinear response of soil nematode was also observed in the 3,200-km east-west-oriented grassland transect in northern China (40). The possible reasons are (i) the increase of aridity caused the type and biomass of aboveground vegetation to decrease or even disappear (15), which disrupted nutrient exchange with the underground biosphere; (ii) different nutrient inputs specifically increased or decreased the diversity and number of subsurface microorganisms, leading to an imbalance or even decoupling of microbial-mediated soil nutrient cycling (19, 20, 42); and (iii) the decrease of soil nutrient availability accelerated competition among microorganisms (45). Interestingly, we found the increase of community dissimilarity (NMDS1) in topsoil was faster than that in subsoil for abundant taxa, while the opposite trend was found in rare taxa under higher aridity stress. This may be explained by the higher turnover in the more nutrient-heavy topsoil due to the wider resource utilization capacity of the abundant taxa, whereas nutrient impoverishment in the subsoil may stimulate rare taxa to increase functional redundancy and resistance to aridity by accelerating turnover (9, 48).

### Sharper ecological abrupt adaption of abundant bacteria taxa to aridity stress.

In our study, abundant taxa showed stronger phylogenetic signals for ecological preferences than rare taxa, which indicated that related species in abundant taxa have more similar ecological preferences over environmental gradients. Recent studies in a variety of ecosystems (e.g., terrestrial and aquatic) support our findings (10, 49, 50). Those studies have suggested that ecological preferences in evolutionary history determined traits and speciation (31, 39, 51). Evolutionary preferences for salinity (52), high temperature (53), and substrate (54) also supported this insight. Microbial responses to environmental disturbances (water addition, carbon addition, and drought) and ecological preferences appear to be phylogenetically conserved across the tree of life (31, 55–57). Thus, the stronger phylogenetic signal for abundant taxa may explain the greater phylogenetic niche conservatism in abundant taxa with an evolutionary history of environmental adaptation. Most importantly, our results indicate the depth of phylogenetic conservatism was strongly positively associated with increasing aridity and drastically increased under higher aridity stress both for abundant and rare taxa. The abrupt, phylogenetically conserved pattern of response to increasing aridity may be explained by decoupling the historical ecological preferences of species based on functional traits and soil nutrients caused by increasing aridity. The negative correlation between functional redundancy index and increasing aridity supported our conjecture for both abundant and rare taxa.

### Assembly processes and species coexistence in abundant and rare taxa along aridity threshold.

Understanding the community assembly mechanisms to increasing aridity is crucial to revealing the stability and tolerance of biodiversity and function for future climate change (11, 15, 20). Based on null model analysis, our results suggested that aridity threshold strongly mediated the dynamic balance of community assembly for abundant and rare taxa. The overriding effect of climate factors (e.g., aridity and MAP) on soil microbials was consistent with other findings in dryland ecosystems (12, 40, 58, 59). Equally important, we found that community assembly exhibited a distinct pattern of nonlinear responses to increasing aridity. Although the coexistence networks based on Spearman correlation may be biased for compositional data (compared with SparCC) (60), the networks can still meet the overall trend of coexistence relationships (61–63). Specifically, the relative influence of stochastic processes on rare taxa and deterministic processes on abundant taxa increased with increasing aridity under lower aridity stress. As aridity continued to increase, both abundant and rare taxa were strongly influenced by deterministic processes. One possible explanation is that rare taxa exhibit stronger stochastic dispersal than abundant taxa under weaker stress pressures (64). Furthermore, the higher diversity of rare taxa also means a more stochastic assembly process (65). When spatial isolation or temporal succession is strong enough, greater environmental heterogeneity enhances the relative influence of deterministic processes on community assembly (22, 66).

In addition, we found that the node-level topological features of abundant taxa decreased significantly with increasing aridity, while the topological features of rare taxa were not significant under lower aridity stress. Both increased rapidly under higher aridity stress, indicating that the abundant taxa were strongly filtered across the aridity gradient, while rare taxa were only selected by the environment under higher aridity stress. We observed faster response rates of abundant taxa with increasing aridity than that of rare ones, indicating that abundant taxa are more sensitive to the intensification aridity than rare taxa. Thus, abundant species could act as the indicators for predicting the response of ecosystem attributes to aridity extremes, and rare taxa might be the microbial seed bank to enhance the resistance or resilience of soil microbiota and play a potential role in maintaining ecosystem stability under serious global aridification. This might be the ecological implications for the investigation of aridity threshold for rare and abundant taxa. It must be acknowledged that our results found the critical aridity level for soil bacterial community collapse to be >0.9, which is higher than the threshold of soil fungus disruption (aridity levels of >0.7) based on global data sets in the recent report (15). This may be due to the close association between soil and plant communities that increases the sensitivity of fungi to drought stress (67, 68). In addition, a recent report (20), combining field study and microcosm experiments, showed that the positive relationship between soil microbial diversity and soil multifunctionality was dominated by soil microbial diversity rather than plant diversity at aridity levels of >0.8. This result might explain the higher tolerance of soil bacteria due to less vegetation coverage in extremely arid areas (northwest of Hexi Corridor) to increasing aridity. Moreover, huge microclimatic heterogeneity may also provide an important contribution to the results. Considering these differences, future studies should focus more on the small variations caused by high environmental heterogeneity at local scale and adopt strategies tailored to local conditions to cope with soil diversity and ecosystem service disruption from increasing aridity.

### Conclusions.

To the best of our knowledge, we systematically provided the first statistical evidence of abrupt adaptation of species coexistence, ecological process, and niche conservation for abundant and rare soil bacteria triggered by diversity to abrupt increases in aridity. Our results underscore that abundant microbial taxa show better ecological adaptation than rare taxa in terrestrial ecosystems to increases in aridity. This finding has important implications for understanding the impact of aridity on the structure and function of abundant and rare soil taxa and how diversity maintenance is associated with soil microbiota responding to global change. Eventually, the abrupt threshold of soil bacteria found can be used for buffering and for building effective adaptation and mitigation measures aimed at maintaining the capacity of drylands for basic ecosystem functioning.

## MATERIALS AND METHODS

### Soil sampling and DNA processing.

Soil samples were collected along the Hexi Corridor in the northwestern portion of Gansu Province and to the west of the Yellow River in China (94°37′ to 103°31′E, 36°56′ to 40°34′N), as described by Jiao et al. (69). In total, 266 soil samples were collected from five habitats (37 agricultural field, 28 forest, 15 wetland, 27 grassland, and 25 desert) and two corresponding soil layers (surface with a depth of 0 to 15 cm and subsurface with a depth of 15 to 30 cm) throughout the transect intervals of 1,257.6 km. Zea mays L. (agricultural field), *Calligonum* spp., *Stipa* spp., *Leymus* spp., *Achnatherum* spp. (wetland, grassland, and desert), and *Populus* spp. (forest) cover most of these habitats. Long-term wind erosion and aridity caused by semiarid climate has formed a soil with loose structure and low organic matter in the Hexi Corridor.

The terrain properties of every site, including longitude (Lon), latitude (Lat), and elevation, were recorded using a handheld GPS unit (eTrex Venture; Garmin, Olathe, KS, USA). MAT and MAP (mean annual temperature [MAT] and mean annual precipitation [MAP]) data for each sampling site were obtained from a national climate database (http://data.cma.cn). Aridity index (mean annual precipitation/mean annual potential evapotranspiration) was extracted from the CGIAR-CSI Global-Aridity and Global-PET database (70). Aridity is presented in our study as one minus the aridity index.

Soil physicochemical characteristics, including soil pH, total organic carbon (SOC), dissolved organic carbon (DOC), microbial biomass carbon (MBC), total nitrogen (TN), ammonia-nitrogen (NH_4_), nitrate-nitrogen (NO_3_), microbial biomass nitrogen (MBN), total phosphorus (TP), available phosphorus (AP), total potassium (TK), available potassium (AK), and cation exchange capacity (CEC), were measured through standard testing methods, as described previously (71). Total DNA was extracted from soil samples (0.5g) using a FastDNA spin kit for soil (MP Biochemicals, Solon, OH, USA). For bacterial diversity, the forward primer 515F (GTGCCAGCMGCCGCGG) and the reverse primer 907R (CCGTCAATTCMTTTRAGTTT) (72) were used to amplify the V4-V5 region of the 16S rRNA gene. The sequences were assigned to their corresponding samples according to the barcode and then quality trimmed with a threshold of average quality scores of higher than 20. Chimera detection and removal were accomplished using the USEARCH tool in the UCHIME algorithm (73). Paired-end sequences that passed quality control were joined, clustered into operational taxonomic units (OTUs) using a 97% identity level cutoff, and assigned to taxonomic groups by SILVA database (release 128) (39). Counts of individual OTUs were scaled by the total number of reads in each sample to account for sequencing biases using the R package DESeq2 (74). In total, 15,429,528 reads were collected, from which 25,981 OTUs were obtained. Neither the α-diversity nor β-diversity of rare and abundant subcommunities differed significantly between surface and subsurface soils in any of the biomes (data not shown); therefore, we did not consider differences between surface and subsurface soils in further analyses.

### Statistical analysis.

OTUs that contained fewer than 20 reads were removed to avoid random effects on the identification of rare taxa (10). To account for different sequencing depths, samples were rarefied to 20,000 sequences each. The division of abundant and rare OTUs was performed as previously described (10, 75). Concisely, OTUs with relative abundances above 0.1% were identified as abundant, while those with relative abundances below 0.01% of the total sequences were designated rare.

### Threshold detection.

Thresholds can occur only if nonlinear regression is a better fit for the data. We considered a threshold the point in aridity when a given variable sharply changes its value (breaking point) (15). Thus, for variable models that fit better with a secondary model than a linear model, we took segmented regression. The “piecewise” function in the “Sizer” package of R (76) was used to identify the threshold and fit OLS (ordinary least squared) and segmented regressions with the threshold. Biodiversity is known as a critical determinant of ecosystem functioning (77). To explore the correlation between biodiversity loss and ecosystem functional changes, we took the value of aridity causing a change in microbial alpha diversity as a segmenting point for other subsequent variables.

### Phylogenetic niche conservatism.

We applied Blomberg's K statistics to characterize the depths of trait conservatism for aridity response in abundant and rare bacterial taxa, as calculated with the “multiPhylosignal” function in the “picante” package of R. The K values quantified the correlation between species and phylogeny, similar to the estimation of phylogenetic intensity of Brownian motion: higher K values (>1) mean deeper phylogenetic signals and niche conservatism, whereas lower K values (≈0) indicate a convergent or random pattern of evolution. In addition, niche breadths of abundant and rare taxa were calculated with the “niche.width” function in the “spaa” package of R according to Levins (78, 79).

### Construction of co-occurrence network.

Co-occurrence networks of abundant and rare taxa were constructed to evaluate the coexistence of species at different aridity thresholds. Spearman's correlation coefficients greater than 0.6 and adjusted *P* values for multiple testing using false discovery rate of less than 0.01 were used to construct these networks. To describe the topology of the networks, we calculated a set of metrics, average degree, average path length, clustering coefficient, density, diameter, heterogeneity, and centralization, through the “microeco” package (80). All networks were visualized using the interactive Gephi platform (81).

### Random forest modeling.

We identified the factors driving the change of microbial diversity and community assembly dynamics using one or both of the following methods: (i) we determined the Spearman's correlation between the microbial diversity in abundance, habitat type, soil depth, and environmental factors, and (ii) RF analysis was applied to identify the main factors including soil and climate variables influencing the microbial diversity and community assembly process. The mean squared error (MSE) values of each variable were sorted to characterize the relative importance, and the larger values indicated higher importance (82). Finally, the variation and significance of the model were determined by generating 1,000 random permutations by the “a3” function in the “A3” package of R.

### NST modeling.

Normalized stochasticity ratio (NST) was utilized to quantify the relative importance of responding to increases in aridity in the process of community assembly dynamic of abundant and rare taxa (23). The NST index used 50% as a critical value to determine whether the assembly process is more deterministic (<50%) or more random (>50%).

### Predicting function of bacteria.

The functional redundancy index (FRI) was used to describe functional profiling of rare and abundant taxa. The absolute FRI was based on the proportion of abundant and rare taxa with a predicted function using the ‘‘FAPROTAX” package in R (83).

### Data availability.

The raw sequence data reported in this paper have been deposited in the Genome Sequence Archive (36) and in the Beijing Institute of Genomics (BIG) Data Center (37), Chinese Academy of Sciences, under BioProject accession no. PRJCA004036 and are publicly accessible at http://bigd.big.ac.cn/gsa.

10.1128/mSystems.01309-21.7 FIG S7 Changes of relative abundances of most dominant phyla at both ends of the aridity threshold for abundant and rare taxa. Download FIG S7, TIF file, 1.5 MB. Copyright © 2022 Pan et al. 2022 Pan et al. https://creativecommons.org/licenses/by/4.0/ This content is distributed under the terms of the Creative Commons Attribution 4.0 International license.
