# Supplementary figures and images for: Aridity Threshold Induces Abrupt Change of Soil Abundant and Rare Bacterial Biogeography in Dryland Ecosystems
Source: mSystems. 2022 Feb 8;7(1):e01309-21. doi: 10.1128/msystems.01309-21 (PMC8823291; doi:10.1128/msystems.01309-21)

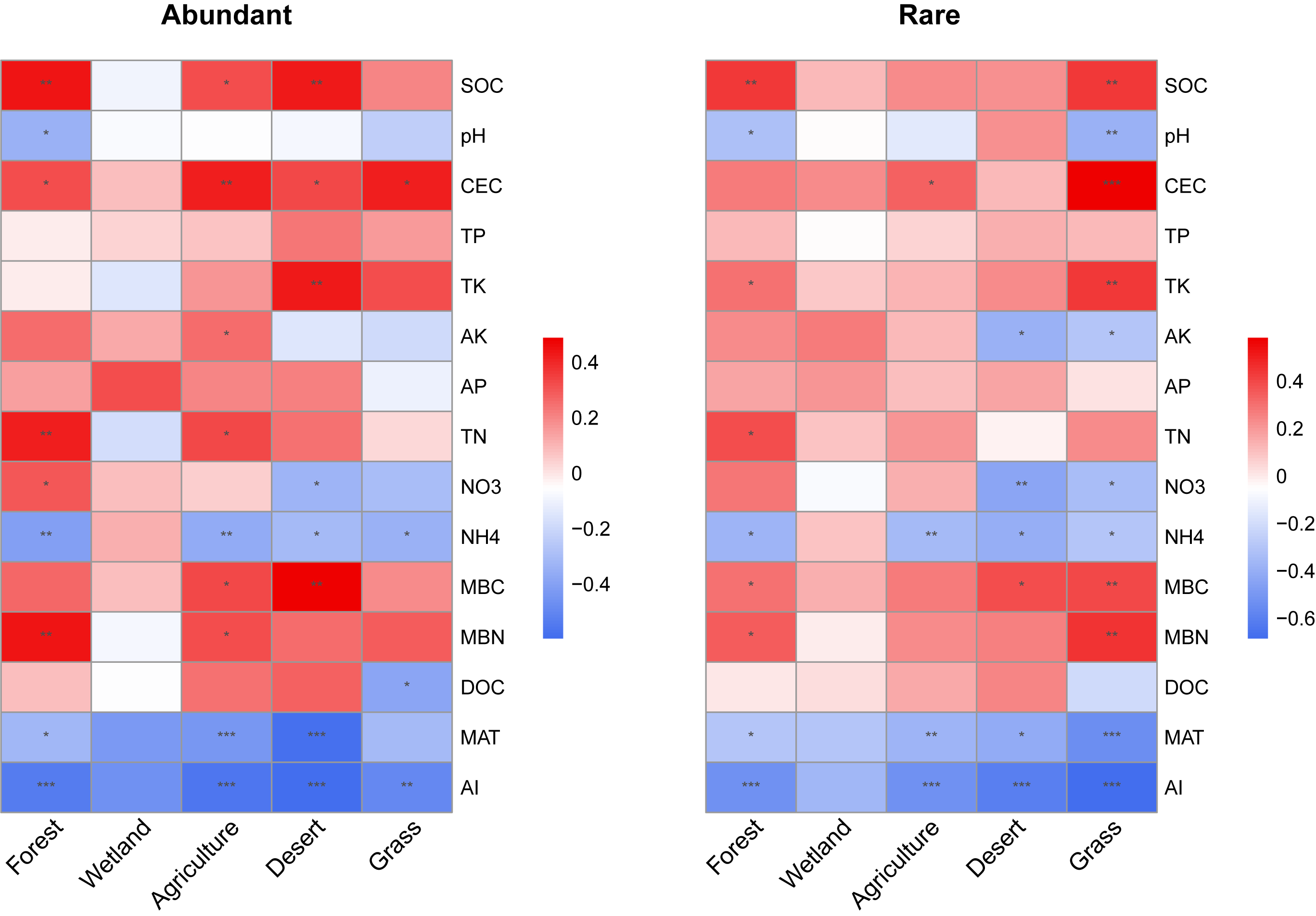

Supplement: FIG S1 [file msystems.01309-21-sf001.tif]

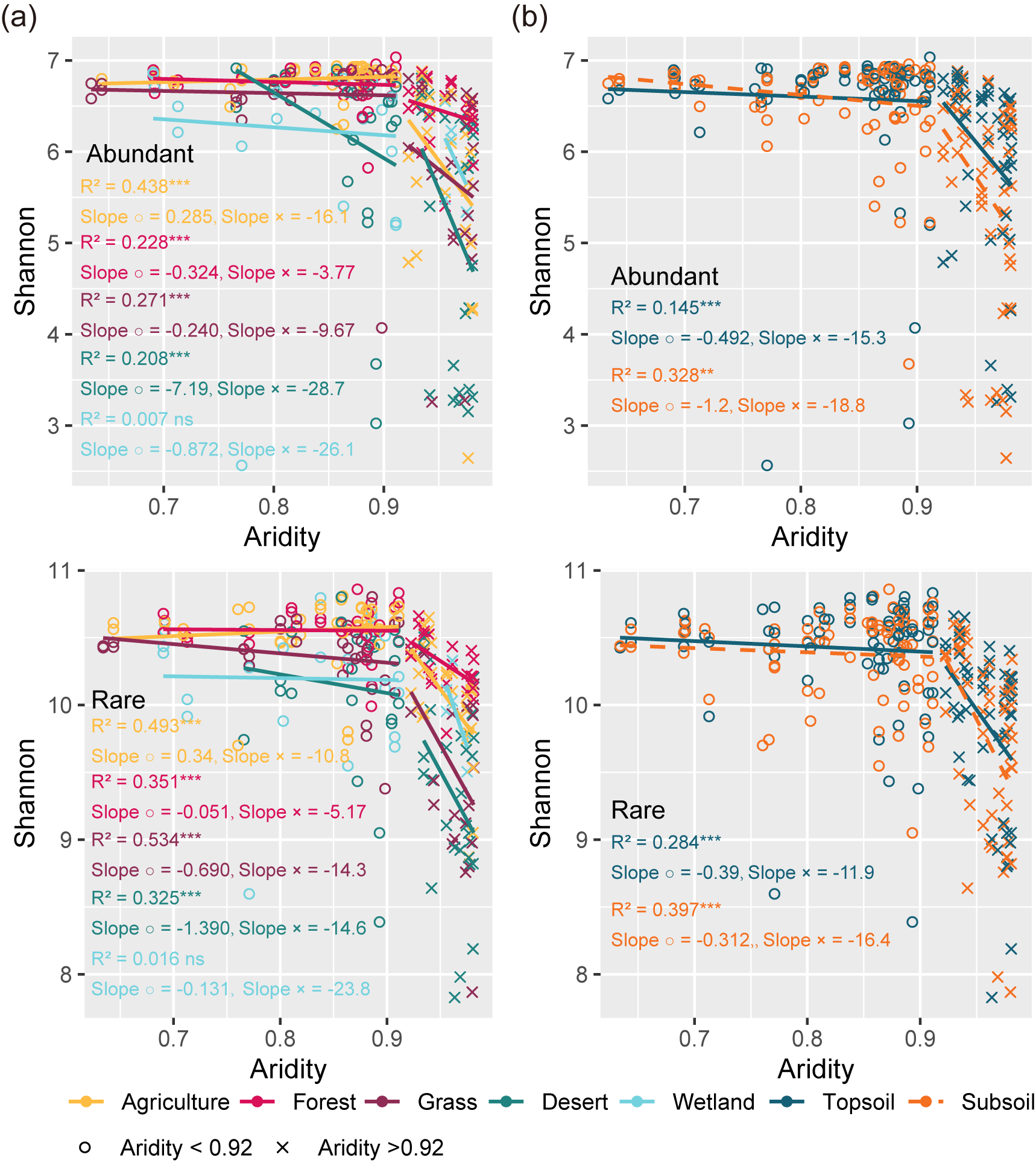

Supplement: FIG S2 [file msystems.01309-21-sf002.tif]

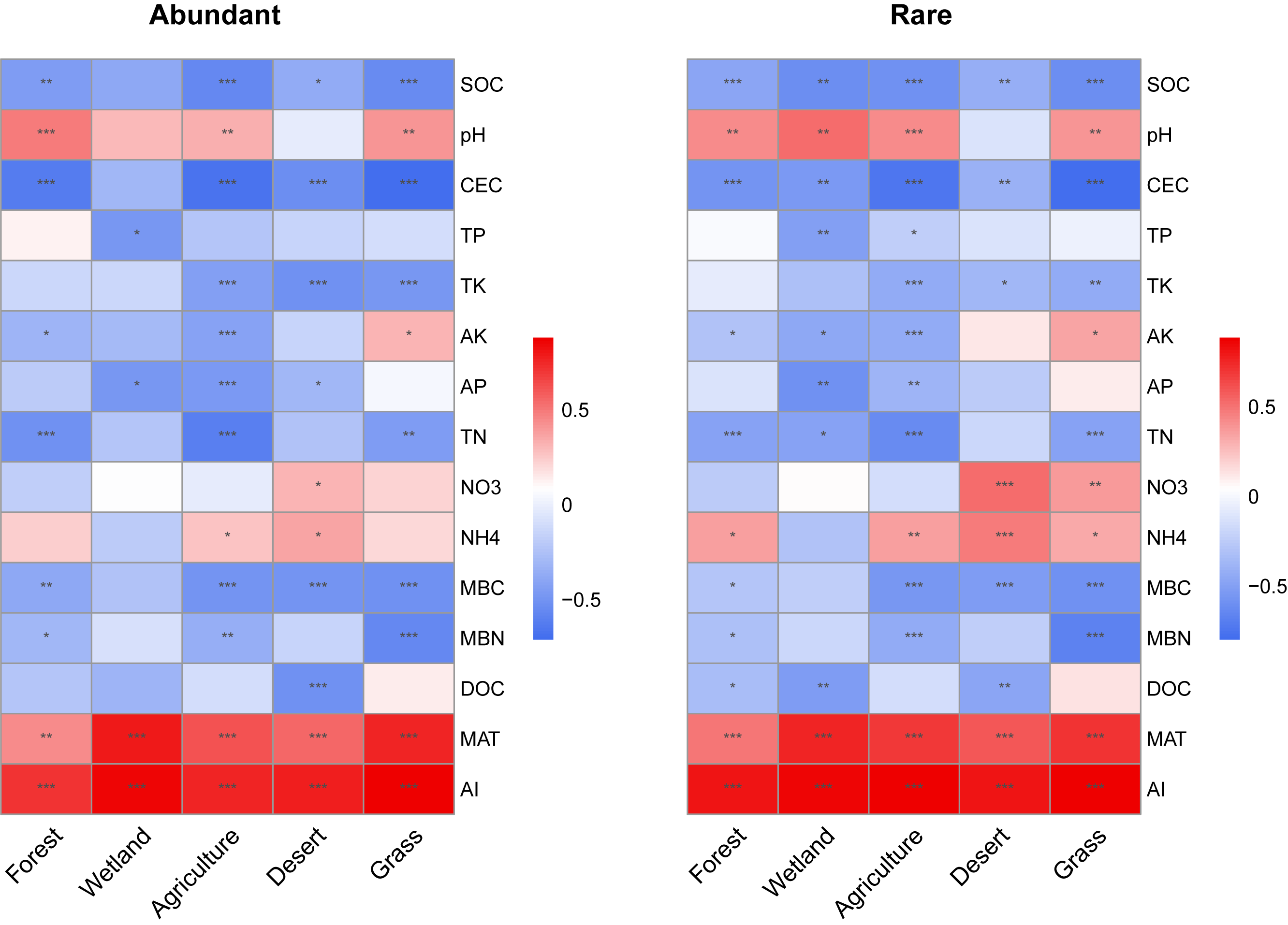

Supplement: FIG S3 [file msystems.01309-21-sf003.tif]

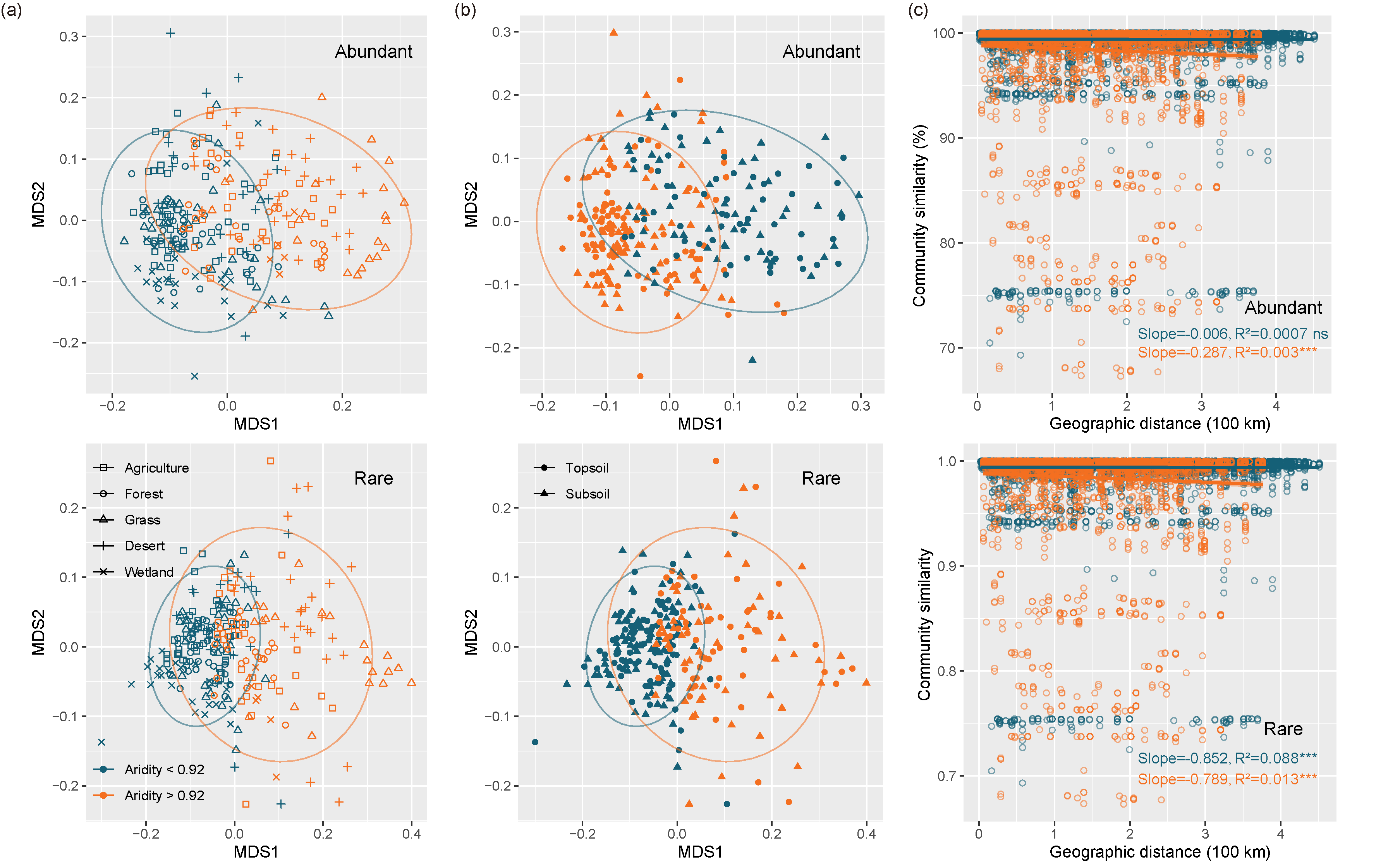

Supplement: FIG S4 [file msystems.01309-21-sf004.tif]

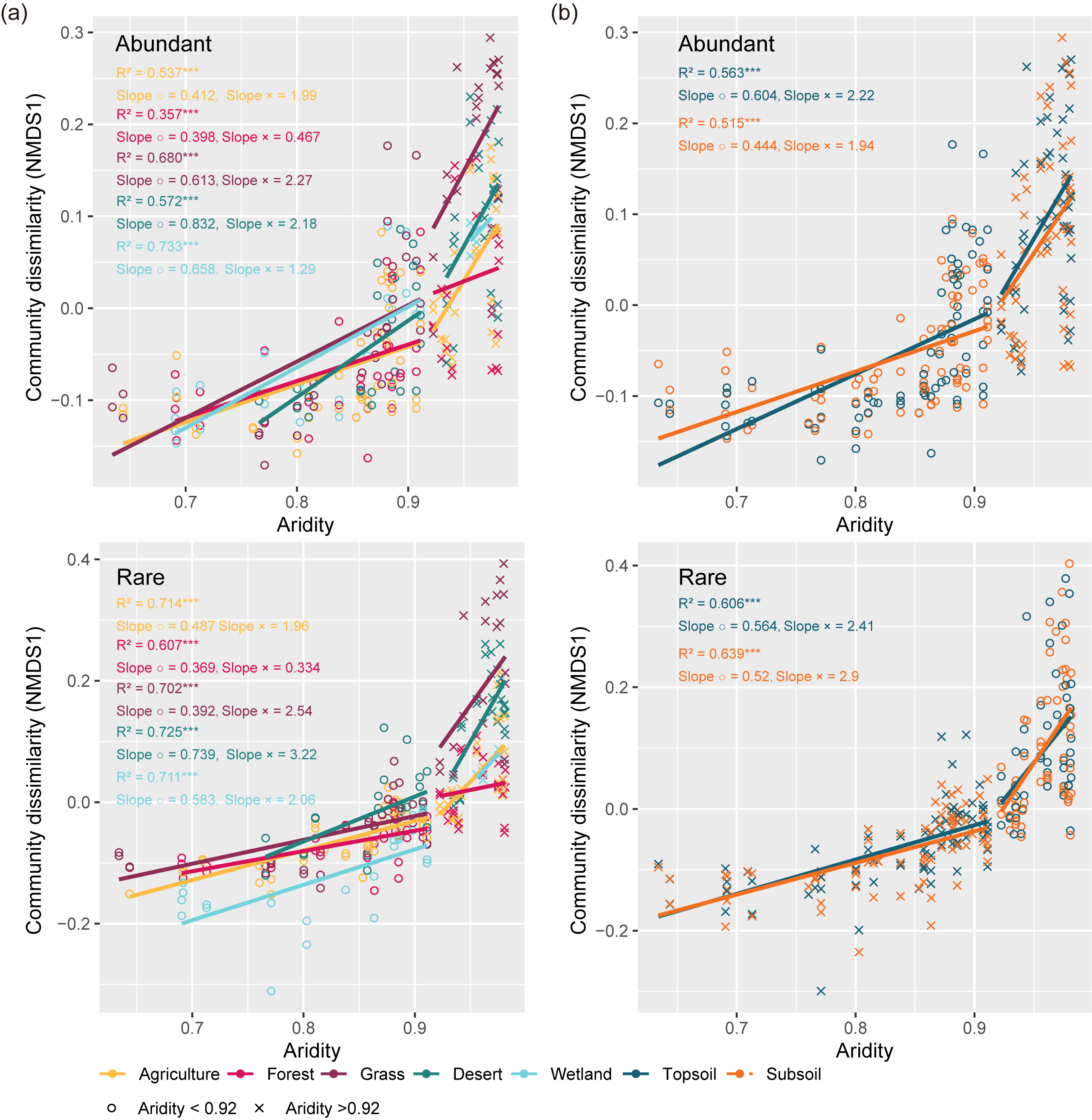

Supplement: FIG S5 [file msystems.01309-21-sf005.tif]

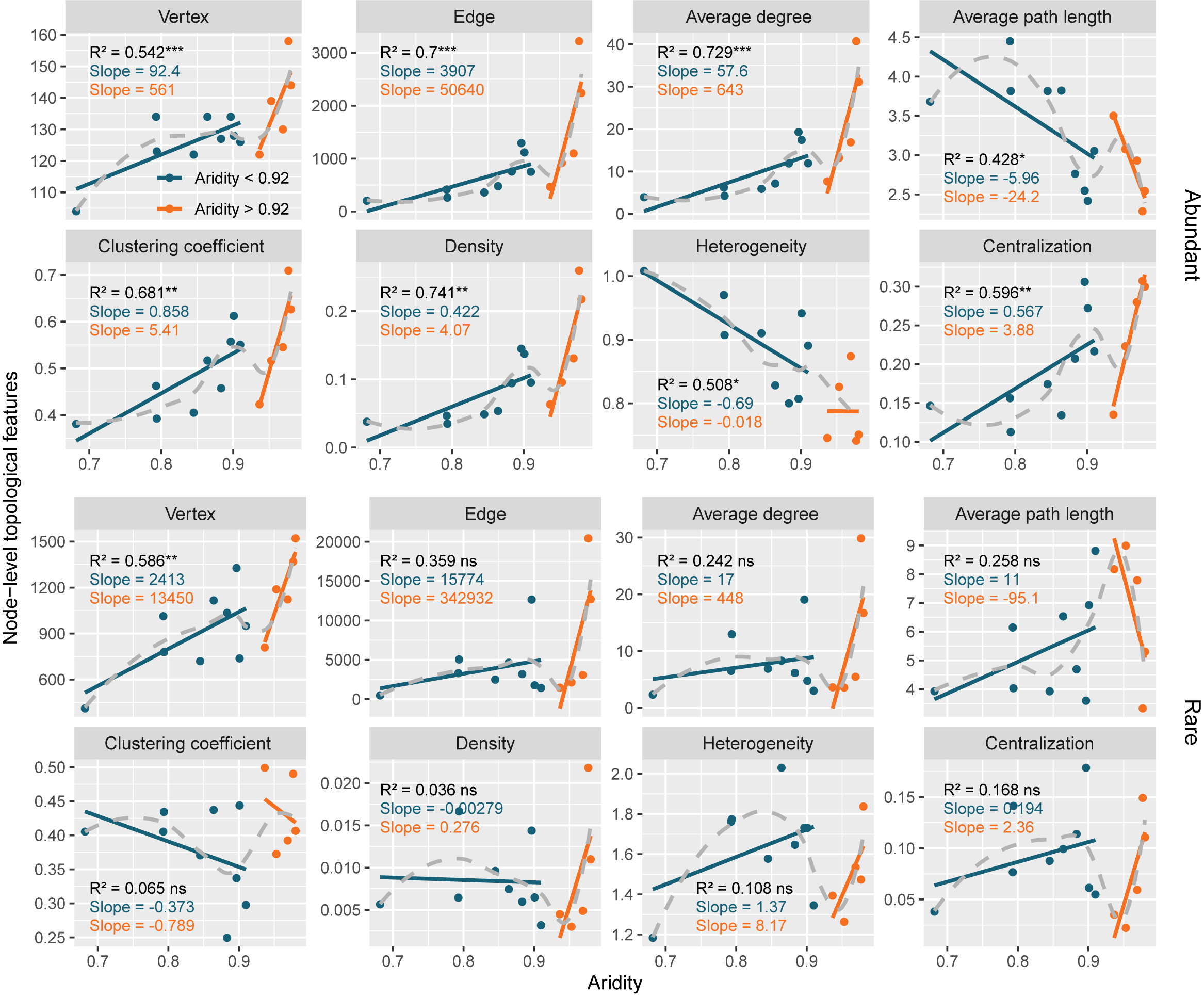

Supplement: FIG S6 [file msystems.01309-21-sf006.tif]

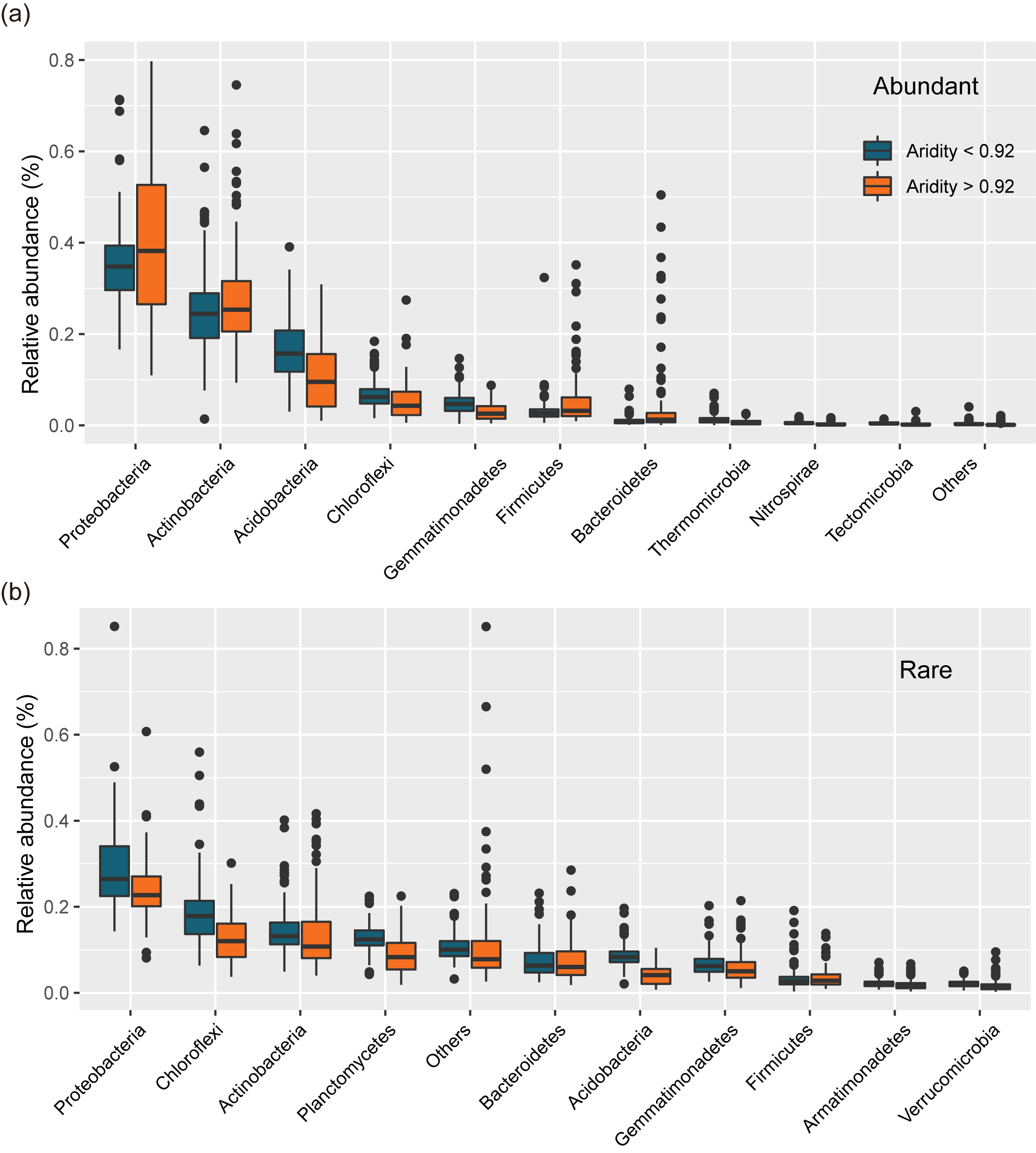

Supplement: FIG S7 [file msystems.01309-21-sf007.tif]
